# Isoflurane-Induced Burst Suppression Is a Thalamus-Modulated, Focal-Onset Rhythm With Persistent Local Asynchrony and Variable Propagation Patterns in Rats

**DOI:** 10.3389/fnsys.2020.599781

**Published:** 2021-01-12

**Authors:** Qianwen Ming, Jyun-You Liou, Fan Yang, Jing Li, Chaojia Chu, Qingchen Zhou, Dan Wu, Shujia Xu, Peijuan Luo, Jianmin Liang, Dan Li, Kane O. Pryor, Weihong Lin, Theodore H. Schwartz, Hongtao Ma

**Affiliations:** ^1^Department of Neurology, The First Hospital of Jilin University, Changchun, China; ^2^Department of Anesthesiology, New York-Presbyterian Hospital/Weill Cornell Medicine, New York, NY, United States; ^3^Department of Radiology, The First Hospital of Jilin University, Changchun, China; ^4^Department of Pediatrics, The First Hospital of Jilin University, Changchun, China; ^5^Department of Neurological Surgery and Brain and Mind Research Institute, Weill Cornell Medicine of Cornell University, NewYork-Presbyterian Hospital, New York, NY, United States

**Keywords:** general anesthesia, burst suppression, widefield calcium imaging, thalamocortical interactions, traveling wave, slow oscillations

## Abstract

**Background:** Inhalational anesthetic-induced burst suppression (BS) is classically considered a bilaterally synchronous rhythm. However, local asynchrony has been predicted in theoretical studies and reported in patients with pre-existing focal pathology.

**Method:** We used high-speed widefield calcium imaging to study the spatiotemporal dynamics of isoflurane-induced BS in rats.

**Results:** We found that isoflurane-induced BS is not a globally synchronous rhythm. In the neocortex, neural activity first emerged in a spatially shifting, variably localized focus. Subsequent propagation across the whole cortex was rapid, typically within <100 milliseconds, giving the superficial resemblance to global synchrony. Neural activity remained locally asynchronous during the bursts, forming complex recurrent propagating waves. Despite propagation variability, spatial sequences of burst propagation were largely preserved between the hemispheres, and neural activity was highly correlated between the homotopic areas. The critical role of the thalamus in cortical burst initiation was demonstrated by using unilateral thalamic tetrodotoxin injection.

**Conclusion:** The classical impression that anesthetics-induced BS is a state of global brain synchrony is inaccurate. Bursts are a series of shifting local cortical events facilitated by thalamic projection that unfold as rapid, bilaterally asynchronous propagating waves.

## Introduction

Burst suppression (BS) is an EEG rhythm characterized by alternating high amplitude electrical discharges separated by variable periods of low electrical activity with a duration on the order of seconds (Swank and Watson, 1949). BS is a dominant brain rhythm under deep general anesthesia (Derbyshire et al., 1936; Brown et al., 2010; Hagihira, 2015; Purdon et al., 2015). Anesthetic-induced BS has long been studied for its effects on neural metabolism and as a potential agent for neuroprotection (Doyle and Matta, 1999; Engelhard et al., 1999; Sanders et al., 2005; Ching et al., 2012). Recent resurgence of interest in the clinical community emerged from its hypothetical association with perioperative neurocognitive disorders, particular with post-operative cognitive dysfunction (Fritz et al., 2016, 2018; Wildes et al., 2019).

Surface EEG recordings have revealed that transitions between burst and suppression appeared to be globally synchronous (Akrawi et al., 1996; Urrego et al., 2014; Purdon et al., 2015). Anesthetic-induced BS therefore has been classically described as a state of global brain synchrony (Clark and Rosner, 1973; Akrawi et al., 1996; Liu et al., 2011; Urrego et al., 2014). However, this concept has been recently challenged (Bojak et al., 2015). Theoretical studies have predicted that the transition from suppression to burst may arise focally within the cortex. Subsequent rapid propagation of neural activity over the cortex gives the superficial appearance of global synchrony (Bojak et al., 2015). Clinically, asynchronous BS cycling under anesthesia has been reported in patients with pre-existing brain pathology, such as corpus callosum hemorrhage (Lambrakis et al., 1999; Lazar et al., 1999), refractory focal epilepsy (Lewis et al., 2013), and status epilepticus treated with intravenous anesthetics (Mader et al., 2014). However, whether asynchronous BS also occurs in a brain without pre-existing pathology is not well investigated.

Thalamocortical interactions have been shown to play a major role in modulating EEG dynamics under anesthesia. Potentiation of gamma-aminobutyric acid (GABA) transmission in the thalamus may lead to oscillatory cortical activity during bursts (Ching et al., 2010, 2012); whereas, mechanisms which operate on a slower time scale, including metabolic factors (Ching et al., 2012) and synaptic depression (Liley and Walsh, 2013), may control the switch between burst and suppression. The interplay between the fast and slow processes has been predicted to self-organize into recurrent, semi-periodic cortical traveling waves (Bojak et al., 2015). Although these theories elegantly explain the serial EEG changes as inhalational anesthesia deepens (Bojak et al., 2015; Hagihira, 2015; Purdon et al., 2015), direct evidence of these traveling wave patterns is limited. High resolution, large-scale recording of cortical neural activity with active thalamic manipulation is required to support such predictions.

In this study, we used high-speed widefield calcium imaging to maximally cover bilateral cortices in order to comprehensively study the spatiotemporal dynamics of neural activity during isoflurane-induced BS. We found evidence against the concept of global synchrony. Cortical transition from suppression to burst was initiated at a spatially shifting, variably localized focus. Whole cortex transition was rapidly completed by fast neural waves that propagated throughout both hemispheres, typically within 100 milliseconds. Neural traveling waves recurred in a delta frequency within the burst periods, and some of them only propagated locally, forming complex regional patterns. Wave dynamics were highly correlated between homotopic cortices, and propagation patterns from each hemisphere often mirrored each other. Finally, we confirmed that thalamic activity modulated suppression-to-burst transitions at the cortices, as silencing one thalamus with tetrodotoxin (TTX) shifted the spatial distribution of cortical onset sites away from the TTX-injected hemisphere.

## Materials and Methods

### Experimental Animals

All experimental procedures were approved by the Jilin University Animal Care and Use Committee. General anesthesia of adult male Sprague Dawley rats (200–350 g) was induced with isoflurane. The concentration was subsequently titrated to an anesthesia depth of burst suppression during maintenance, usually within 1–2%, guided by concurrent cortical local field potential (LFP) monitoring (Rampil and Laster, 1992). Body temperature was maintained at 37°C with a regulated heating pad. The heart rate, SpO_2_, and the end tidal carbon dioxide (EtCO_2_) were monitored with a small animal capnography and were sustained throughout the experiment (heart rate: 250–350 pulses per minute). The head was fixed in a stereotaxic frame.

### Calcium Dye Staining

The calcium indicator Oregon Green 488 BAPTA-AM (OGB-1, Life Technologies, Grand Island, NY, United States) was used for widefield recording of neuronal activity. We chose OGB as our calcium indicator (Paredes et al., 2008) because of its ultrashort rise time in comparison to genetic-encoded calcium indicators (Chen et al., 2013), which facilitates differentiating the onset time of bursts across the cortical surface. Convection enhanced delivery was employed to bulk load the entire neocortex with OGB-1 (Ma et al., 2014). In brief, 50 μg of OGB-1 was diluted in 5 μL of DMSO-F127 then in 50 μL of artificial cerebrospinal fluid. 8 μL of OGB-1 solution was injected in the neocortex via a glass electrode (50–100 μm opening) placed ∼1 mm below the brain surface at a rate of 100 nl/min, using a micro-pump (WPI, Sarasota, Florida). A ∼ 5 × 8 mm craniotomy window was then opened over each stained hemisphere between bregma and lambda, and the exposed brain was covered with silicon oil (12,500 centistoke) to preserve cortical moisture.

### Optical Imaging and LFP Recording

A high-speed camera (J-MC023MGSY, Lighting Mind Inc., Changchun, China) using a tandem lens (50 × 50 mm) arrangement was focused 300–400 μm below the cortical surface. A 470 nm LED light (Thorlabs, Newton, NJ, United States) was employed as the illumination source for the calcium-sensitive dye. The illumination was directed using fiber-optic light guides. A 510 nm long-pass filter was placed before the camera to record the calcium fluorescence. Camera frame rate was set to be 40–120 Hz to achieve high temporal resolution in order to study burst onsets and propagation pathways. For each animal, up to 6 of 20-s calcium imaging sessions were performed on each animal to avoid photobleaching and phototoxicity. A glass electrode (50–100 μm tip opening) filled with 0.9% NaCl solution was inserted 300–500 μm below the cortical surface to record LFP. The LFP was amplified, band-pass filtered (1–500 Hz) and digitized at 1000 Hz with bl420E^+^ system (Taimeng Technology Co., Ltd., Chengdu, China). The frame indicators provided by the camera were also recorded by the bl420E^+^ system to synchronize LFP and optical imaging data.

### Data Analysis

The LFPs were used to detect individual bursts (Liou et al., 2019). Candidates of burst were first systematically searched by screening for periods when LFP signals went beyond ±3 root mean square. Each candidate period needed to be more than 500-ms apart to be considered independent. All burst candidates were further visually reviewed, manually merged, or removed by consensus among the researchers.

Optical data were processed and analyzed by customized MATLAB code. The raw data was first spatially convolved with a Gaussian kernel (σ = 3 pixels) to improve the signal-to-noise ratio (S/N). The calcium imaging data was then calculated as dF/F, where F was the baseline fluorescence during suppressions and dF was the signal change during bursts.

The S/N of bursts was examined to ensure data quality. For each pixel, the signal amplitude was defined as the peak amplitude of calcium fluorescence change during bursts and the noise level was defined as the s.d. of the calcium fluorescence trace during the suppressions. Pixels with S/N < 5 were excluded from further data analysis.

The calcium signals of the pixels overlapping with the LFP electrodes were programmatically screened to count the number of calcium waves and measure interpeak intervals. For each burst, local burst onset time at each pixel was defined as the time when its calcium signal reached half of its peak fluorescence intensity during the first calcium wave. The geometric center of the earliest 10% pixels was defined as the cortical burst onset site. Time required to propagate throughout a hemisphere was defined as the duration that from 10 to 90% of pixels reached onset threshold.

Burst onsets and propagation patterns were compared between bilateral hemispheres. Cross-hemisphere delay was defined as the difference in the burst onset time of each hemisphere. Similarity of propagation patterns between bilateral hemispheres was determined by two independent reviewers who were not involved in the data collection. A burst was only considered bilaterally similar if both reviewers considered the propagation patterns of each hemisphere mirroring each other. Burst dynamic similarity between the bilateral hemispheres was compared to intra-hemisphere similarity, quantified by correlation coefficients of calcium signals. The cortical surfaces were therefore divided into 4 quadrants. Pairs of quadrants were categorized according to their topological relations: ipsilateral, homotopic, and remote. Average calcium signals within each pair category were then correlated and compared.

### TTX Injection

To study the effects of thalamic activity on cortical dynamics during isoflurane-induced BS, bilateral calcium imaging was first performed to record the spatiotemporal dynamics of BS at baseline. Then, TTX (0.3 mM, 1 μL in 0.9% NaCl saline) was injected into either of the thalami, which has been previously shown to electrically silence 1–1.5 mm^3^ of the thalamic tissue (Sacchetti et al., 1999). The injections targeted the ventrobasal complex (2.6 mm posterior to Bregma, 2.8 mm lateral to the midline, and 5.8--6.0 mm ventral to the surface of the cortex^1^). The injection pipettes were removed afterward to restore imaging field. Then, simultaneous LFP and widefield calcium imaging were performed. Frequencies of BS cycling during 5 to 15 min before TTX injections were compared to 5 to 15 min afterward.

## Results

### Cortical Burst Onsets Are Focal, Variably Located and Followed by Rapidly Propagating Waves

**FIGURE 1:**
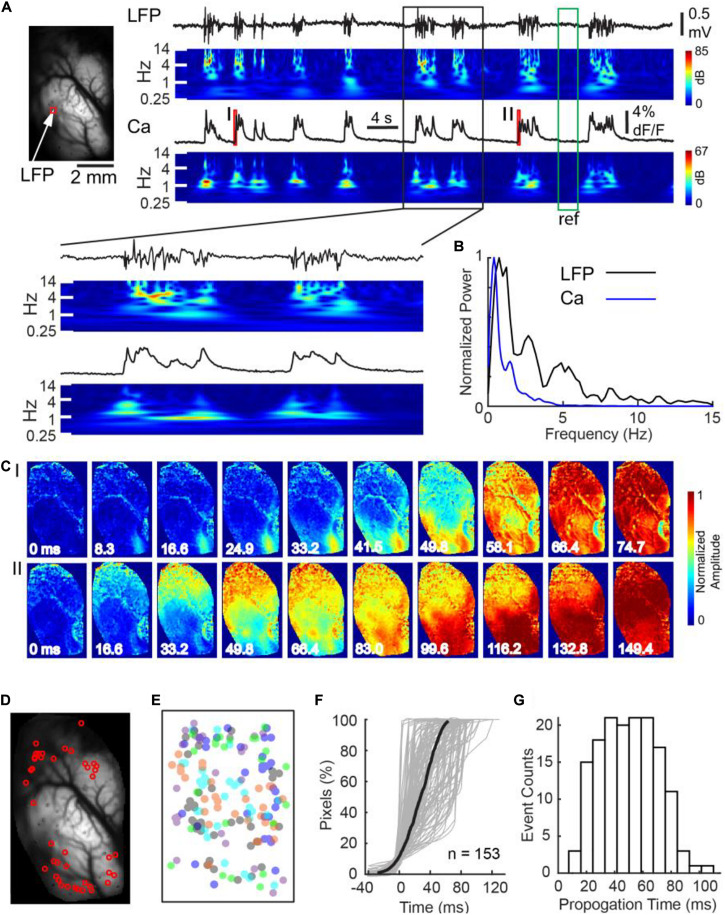
Calcium imaging at burst onset at a single hemisphere. **(A)** Left panel: field of view. The white arrow and red box indicate where the LFP and calcium fluorescence, displayed on the right, were recorded, respectively. Right panel: LFP and calcium fluorescence traces and their corresponding spectrograms. The spectrograms were normalized to the reference period marked by the green box. Calcium dynamics during the onsets of the two bursts, marked with red bars I and II, are further displayed in Panel C. A black box indicates a section of data which was shown with expended time scale in below. **(B)** The power spectrum of Ca and LFP traces during bursts shown in A. **(C)** The spatiotemporal evolution of calcium signals during the two burst onsets. Each burst first emerged at a small focus and subsequently propagated contiguously throughout the whole field of view. Notice the two bursts had separate cortical onset sites. **(D)** The spatial distribution of cortical onset sites of the first wave of 42 bursts recorded in one animal. **(E)** The spatial distribution of cortical onset sites of 153 first waves in five animals. Each color represents one animal. The center of each animal’s imaging window is superimposed on each other. Cortical onset sites did not distribute into any single dominant region. **(F)** Rapid propagation of burst activity over a hemisphere. Percentage of pixels that crossed their burst thresholds was plotted against time. Each gray line represents the recruitment process of an individual burst. The thick black line represents the average recruitment process of 153 bursts in 5 animals. **(G)** The distribution of burst propagation time.

Adult male Sprague–Dawley rats were anesthetized with isoflurane, titrated based on the cortical LFP to achieve an anesthesia depth that produced a BS rhythm. A total of 153 bursts in 5 animals were successfully recorded with concurrent cortical LFP recording and unihemispheric widefield calcium imaging (Figure 1). On average, there were 24.1 ± 5.2 bursts per minute, with each burst lasting 1.26 ± 0.61 s (all data presented in this manuscript as mean ± s.d). The mean LFP amplitude of the bursts was 0.67 ± 0.21 mV. The onsets of LFP discharges coincided with a sudden surge in calcium fluorescence at the electrode-sampled cortex (Figure 1A). Both of the power spectral density of LFP and calcium traces showed concentrated power in the slow-delta range (Figure 1B).

To test whether the onset of bursts exhibited global synchrony, we compared each pixel’s burst onset time in the first wave of each burst, defined as when its calcium signal reached half of its maximum. We found a significant variation of burst onset time across the hemispheric surface (Figures 1C–E). Calcium signals first emerged at a cortical focus, termed the cortical onset site, and subsequently propagated across the whole field of view (Figure 1C). The cortical onset sites, however, were not restricted in a single region (Figures 1C,D). Analysis of 153 bursts in 5 animals showed that their spatial distribution appeared non-ordered (Figure 1E). Despite of focal onsets, subsequent propagation of calcium signal across the hemispheric surface was contiguous and rapid, giving the superficial resemblance of global synchrony. Average temporal delay from 10 to 90% of the field of view reaching the onset thresholds was 53.8 ± 24.9 ms (*n* = 153 bursts; Figures 1F,G).

### Bursts Consist of Multiple Propagating Waves With Variable Degrees of Propagation

**FIGURE 2:**
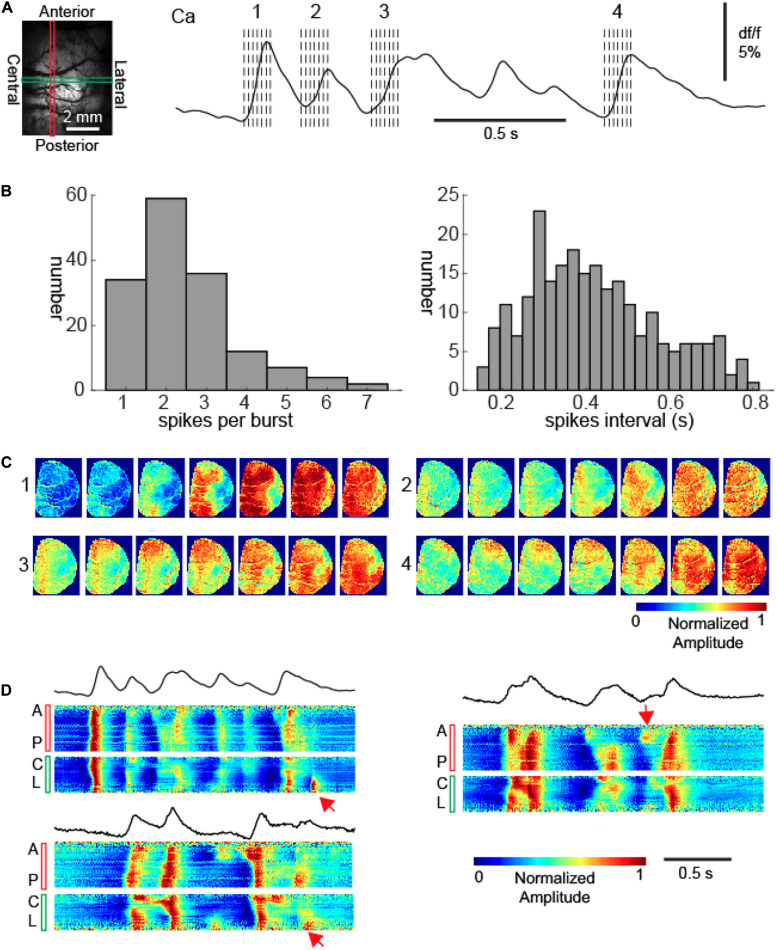
Recurrent calcium waves with asynchrony during bursts. **(A)** Left: the field of view. A vertical red rectangle and a horizontal green rectangle represent two intersecting linear regions of interest (LROI). The calcium signals from each of these LROIs are further displayed in Panel **(D)**. Right: the calcium signal recorded from the intersection of the two LROIs. The spatiotemporal evolution of four calcium waves are further displayed in Panel **(C)**. The dashed lines indicate the timing of images displayed in Panel **(C)**. **(B)** Left: the distribution of waves per burst. Right: the distribution of inter-wave intervals within bursts. **(C)** The propagation patterns of four calcium waves within a burst. Notice the variation of cortical onset sites and propagation patterns. **(D)** The calcium signals from the LROIs. Three bursts are displayed, with the upper left one corresponding to the burst displayed in Panel **(A)**. The top black traces: the average calcium signals of all pixels. The bottom color panels show the vertical anterior-posterior (A-P) and horizontal central-lateral (C-L) propagation of calcium waves from the LROIs. The red arrows provide examples where waves propagated only locally.

Each burst typically consisted of more than one calcium wave (Figure 2A). Among the 153 bursts, we observed 412 calcium waves at the electrode-sampled cortex, with each burst containing 2.69 ± 1.30 waves with an average interpeak interval 0.42 ± 0.16 s (Figure 2B, 1.72–3.84 Hz). If the waves were coherent across the cortical surfaces within each burst, in other words, if the propagation pattern was conserved from the first to the subsequent waves, the neural activity would still be considered globally synchronous.

However, that was not the case; each subsequent wave of an individual burst had a unique site of onset and propagation pattern in comparison to the first wave (Figure 2C). Indeed, subsequent waves exhibited dynamic variability and spatial complexity (Figure 2D). Although the first waves generally propagated across the entire imaged cortices, subsequent waves often aborted their propagation and therefore were spatially limited (Figure 2D). While the initial wave in each burst (*n* = 153) propagated across the entire hemisphere, 36.7% (*n* = 95/259) subsequent waves displayed limited propagation (chi-square test, *p* < 0.001).

### Burst Propagation Sequences Are Preserved Between Hemispheres

**FIGURE 3:**
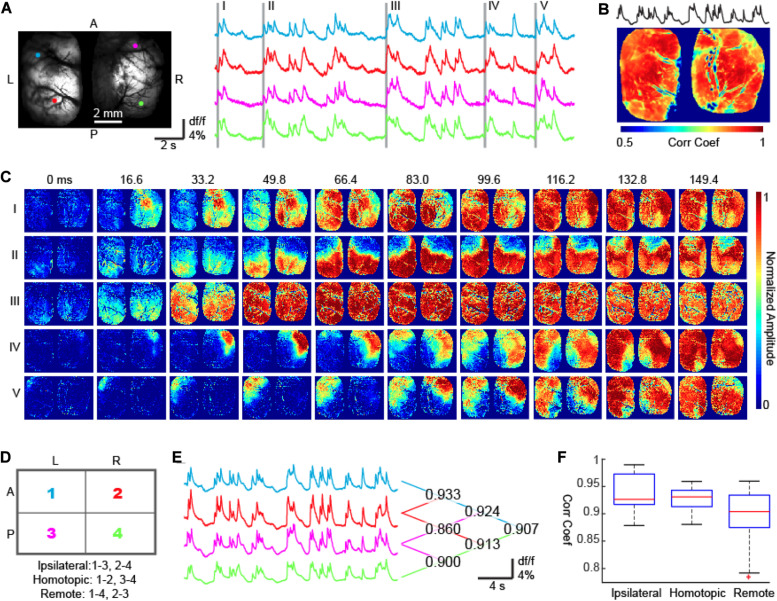
Bi-hemispheric calcium imaging of bursts. **(A)** The field of view. Four colored circles show 4 ROIs whose calcium signals are shown on the right. The first waves of five bursts are highlighted with gray bars. A, Anterior; P, Posterior; L, Left; R, Right. **(B)** The correlation coefficient map between the calcium signals recorded from each pixel and the averaged calcium signal of all pixels. Corr Coef: Correlation Coefficient. **(C)** The onset and propagation of five bursts. Burst I: unilateral onset with non-mirrored propagation. Bursts II and III: bi-hemispheric onsets and mirrored propagation. Bursts IV and V: unilateral onset with mirrored propagation. **(D)** The bilateral hemispheres were divided into 4 quadrants, which formed 3 categories of quadrant pairs according to their geometric relationships: ipsilateral, homotopic, and remote. **(E)** Averaged calcium fluorescence of the 4 quadrants. The correlation coefficients between any 2 of the 4 signals were reported on the right. **(F)** A MATLAB-generated box plot of signal correlation of quadrant pairs. One-way ANOVA test (*p* < 0.001, *F* = 29.54, degree of freedom = 27) and Tukey–Kramer *post hoc* analysis indicates that the correlation coefficient between ipsilateral quadrants and homotopic quadrants are comparable (*p* = 0.544) and significantly higher than that between remote quadrants (*p* < 0.001 for both IS and HS).

Next, we compared burst propagation patterns between the two hemispheres. LFPs with concurrent bi-hemispheric widefield calcium imaging were recorded in a separate group of 5 rats (*n* = 170 bursts). We found a high degree of interhemispheric synchrony (Figure 3A). The average correlation coefficient between the average calcium signal and individual pixels was 0.848 ± 0.112 (*n* = 26641 pixels), again giving an impression of bilateral synchrony (Figure 3B). However, using a finer time scale, we detected temporal delays between the hemispheres and unilateral onsets for each event. Among the 170 recorded bursts, 40% (*n* = 68) had focal onset in the right hemisphere, 37.6% (*n* = 64) had focal onset in the left hemisphere and 22.4% (*n* = 38) had had ambiguous laterality (Figure 3; chi-square test, *p* < 0.001). Cross-hemisphere delay was 64.35 ± 52.39 ms, comparable to the propagation speed within one hemisphere, indicating that, on average, a similar number of synapses needed to be crossed in each scenario (Figure 1E).

Brain lesion studies have shown that the corpus callosum plays an essential role in coordinating burst suppression cycling between the two hemispheres (Lazar et al., 1999). We therefore expected neural activity between homotopic cortices to be tightly locked, generating a similar spatial sequence of burst propagation between the two hemispheres. In agreement with our hypothesis, 138 out of 170 bursts had onset sites and subsequent propagation sequences that mirrored each other in both hemispheres (e.g., Figures 3C-II,III,IV,V); however, there were still 32 out of 170 bursts showing uncorrelated interhemispheric sequences (e.g., Figure 3C-I, chi-square test, *p* < 0.001). Overall, the correlation coefficients of calcium signals between homotopic cortex pairs were comparable with those between neighboring cortices ipsilaterally (Figures 3D–F, see figure legends for statistics).

### Thalamic Activity Modulates Cortical Burst Onsets

**FIGURE 4:**
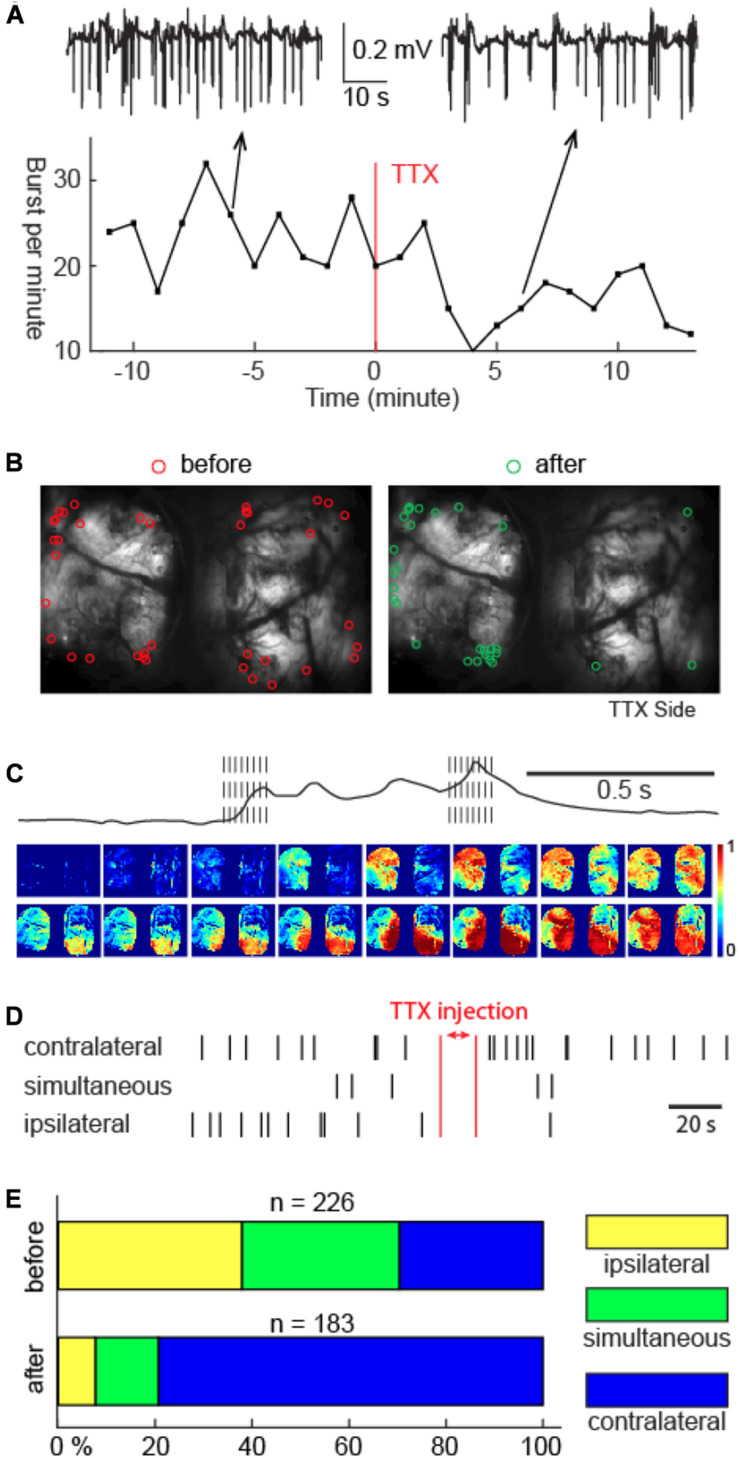
Unilateral thalamic VB complex TTX injection slowed down BS cycling, shifted cortical onset sites, but preserved burst propagation. **(A)** Cortical LFP recordings showed slowdown of BS cycling caused by TTX injection. Top inlets: 1-min LFP recordings 6 min before versus 6 min after TTX injection. **(B)** The spatial distribution of the onset sites before and after TTX injection. TTX was injected in the right thalamus. **(C)** Propagation of one example burst after TTX injection. Note: the spatial continuity of calcium signal propagation across the cortical surface. The propagation pattern of the last calcium wave was different than the first calcium wave. **(D)** Laterality of cortical burst onset sites before versus after TTX injection in one animal. Bilaterally simultaneous onsets are assigned to bursts that have cross-hemispheric delay no greater than 1 frame. **(E)** Summary of the cortical onset laterality study in 5 animals.

Prior electrophysiology studies have shown that >30% of thalamic neurons were active during suppressions compared with <5% of cortical neurons (Steriade et al., 1994). This divergence suggested a differential role of the thalamus and cortex in BS rhythmogenesis. In order to explore the role of the thalamus, we injected TTX unilaterally into the VB complex. The VB complex was chosen due to its dense reciprocal connections with the somatosensory cortex, which constitutes a significant part of our field of view. After unilateral VB complex TTX injection, cycling of BS continued (Figure 4A), although its frequency was modestly reduced (25.44 ± 5.49 to 22.10 ± 5.81 bursts per minute, *n* = 5 animals; *p* = 0.033 two tailed paired *t*-test). However, the spatial distribution of cortical burst onset sites underwent a dramatical change (Figures 4B–E). Instead of evenly distributed between the two hemispheres, TTX injection caused the cortical burst onset sites to shift away from the hemisphere whose thalamus was inactivated (Figures 4B–E, *n* = 226 versus 183 before versus after TTX injection, 5 animals, chi-square test, *p* < 0.001). The onset laterality modulation was not detected in control experiments, during which 0.9% saline was injected into the VB complex (Supplementary Figure 1, *n* = 60 versus 57 before versus after saline injection, 2 animals, chi-square test, *p* = 0.455).

Thalamic TTX injection did not qualitatively change neural propagation patterns once a burst started. Propagation of the subsequent calcium waves remained spatially contiguous in both hemispheres, and, within a burst, multiple calcium waves still displayed individually unique propagation patterns, analogous to that before the TTX injection, as shown in Figure 4C.

## Discussion

Our study refutes the classical hypothesis that anesthetic-induced BS is a state of global brain synchrony. The cortical transition from suppression to burst is a focal event with rapid bilateral propagation. The rapidity of whole cortex recruitment creates a false impression of global synchrony. While onset and directionality of each wave showed marked variability, the spatial sequences of burst propagation remained tightly correlated between both hemispheres. We also found that an intact thalamocortical loop was a major driver for the initiation of bursts. This study provides a clear picture of the spatiotemporal dynamics of anesthetic-induced BS in a mesoscopic scale, filling the knowledge gap between clinical studies, surface EEG recordings, and our understanding of burst mechanisms on a cellular level. Given that BS can arise from a variety of different mechanisms and anesthetics, we cannot necessarily extrapolate our findings to all types BS and our findings may be model- and anesthesia-specific.

Our first key finding was that cortical dynamics were dominated by rapidly propagating waves under deep isoflurane anesthesia. These complex waves, first discovered by wide-field voltage imaging studies (Lippert et al., 2007), have been considered a hallmark of disinhibited cortex (Huang et al., 2004; Pinto et al., 2005; Smith et al., 2016; Liou et al., 2017). This is because unopposed excitation, conducted by cortico-cortical projections, permits a chain reaction in space, emerging as rapid propagating waves (Golomb and Amitai, 1997; Liley et al., 2002; Kramer et al., 2005). In our study, once bursts were triggered, cortical calcium signals arose sequentially in space and recruited both hemispheres rapidly, usually within <100 milliseconds. The existence of neural propagating waves was compatible with the novel idea that inhalational anesthetics, despite traditionally considered as CNS depressants due to their hyperpolarizing effects on individual neurons (Nicoll and Madison, 1982; Patel et al., 1999; Hemmings et al., 2005), may cause paradoxical neocortical hyperexcitation at a circuit level (Kroeger and Amzica, 2007; Ferron et al., 2009). Isoflurane has been shown to preferentially inhibit interneuron activity and inhibitory postsynaptic potential amplitudes in brain slice studies (Ferron et al., 2009). *In vivo* studies showed that cortical responses to thalamic inputs are also enhanced and prolonged (Detsch et al., 2002; Sellers et al., 2015).

Cortical burst onset sites, while not spatially conserved, were more often found at the edges of the imaging window rather than in the center. This should not be interpreted as a preferred region of burst onsets. Due to the curvatures of the cortices, the imaging window could not entirely cover both cortices, particularly the temporal and entorhinal regions. Bursts which first developed at regions outside the imaging window would arrive first at the edge of the imaging window because of their contiguous propagation patterns, therefore increasing the probability that we would find cortical onset sites at the edge of the imaging windows.

Our study showed that a single burst could host multiple cortical propagating waves. The distribution of the interwave intervals fell within a delta range, confirming a rhythmic neuronal depolarization in the superficial cortical layers. Some prior anesthetic studies also showed that brief alpha oscillation may exist in the frontal region within each burst (Purdon et al., 2015). However, this was not identified in our study, which could be caused either by the low-pass effect of the calcium indicator or the source of the optical signals, which arose preferentially from the more superficial cortical levels. Advanced imaging along the z-axis direction and multi-channel depth electrodes would be a future direction of study to clarify this issue.

Within each burst, although the first wave always propagated throughout both cortices, subsequent waves could be spatially restricted. Failure of global propagation suggested that the cortical network gradually lost its excitability after burst onset. This phenomenon has been attributed to synaptic vesicle depletion or glutamate receptor downregulation (Kroeger and Amzica, 2007; Bojak et al., 2015), although adaptative potassium currents can curb network excitability within the same time scale and produce incompletely propagating waves as well (Liou et al., 2020).

Our bilateral propagation study aligns with the prediction of clinical lesion studies that cross-hemisphere burst propagation is mediated by corpus callosum projections (An et al., 1996; Lambrakis et al., 1999; Lazar et al., 1999). Wide-field voltage imaging in rodents with congenital corpus callosum agenesis, as expected, failed to coordinate bi-hemispheric dynamics under both awake and anesthetized states, although the depth of anesthesia was not specifically controlled in that study (Mohajerani et al., 2010).

Our finding that cortical burst onset was a thalamus-driven process aligns with prior clinical observation. Somatosensory inputs were first reported to trigger bursts under deep isoflurane anesthesia (Yli-Hankala et al., 1993). Follow-up studies confirmed that visual (Hartikainen K. et al., 1995) and auditory inputs (Hartikainen K.M. et al., 1995) were also adequate to trigger bursts. In those scenarios, the inputs from the sensory relay nuclei of the thalami provided the initial trigger to the corresponding cortical regions, eliciting a transition from suppression to burst, leading to subsequent rapid propagation across the cortices. Our proposal that cortical bursts are modulated thalamic activity is compatible with *in vivo* intracellular electrophysiology findings that thalamic relay neurons display more autonomous activity than cortical neurons during anesthetics-induced BS (Steriade et al., 1994). Our finding is also supported by a recent human EEG study that neural information flows from the bilateral thalami to the neocortex during BS (Japaridze et al., 2015).

The differential roles of the thalamus and cortex in burst onset were expected as neurophysiological effects of general anesthetics were regionally variant (Christophe et al., 2005; Velly et al., 2007). However, the variability of cortical onset site is intriguing. Random regional fluctuations in cortical cellular energy state (Ching et al., 2012) and synaptic calcium dynamics (Kroeger and Amzica, 2007; Liley and Walsh, 2013) might explain variability in onset location and shifting susceptibility to burst events. On the other hand, the variability of cortical onset sites may result from the propagation directionality and reflect regional variance in thalamic drive. The higher order thalamic nuclei, which are more autonomous and project more widely to the cortex than the primary relay nuclei, may trigger more bursts, resulting in a wide distribution of cortical onset sites (Ramcharan et al., 2005). Although isoflurane-treated brain slices without dedicated preservation of subcortical inputs could still maintain BS cycling with supplementary carbachol (Lukatch et al., 2005), *in vivo* burst generation and cycling could be dominated by interactions between the cortex and thalamus (Steriade et al., 1994).

The brain rhythm presented in this study, by adopting the definition proposed by Steriades et al., can also be labeled as “slow oscillation,” with the neurons cycling between on and off states at a frequency less than 1 Hz (Steriade et al., 1993). Indeed, rodent studies that employed isoflurane to model slow oscillation have revealed similar spatiotemporal properties of cortical dynamics (Sanchez-Vives, 2020), including focal onset followed by cortex-wide propagation waves (Stroh et al., 2013; Aedo-Jury et al., 2020). However, in clinical anesthesiology, “slow oscillation” has been specifically reserved to label an EEG pattern that is qualitatively different from the rhythm presented in this study (Purdon et al., 2015). Caution needs to be taken due to the cross-discipline differences in terminology.

In conclusion, our study provides a comprehensive, mesoscopic view of the spatiotemporal dynamics of isoflurane-induced BS. As studies in anesthesia have provided many unique perspectives in understanding cognition, we believe that advances in understanding anesthetic-induced brain rhythms could have increasing contribute to the field of systems neuroscience.

## Data Availability Statement

The raw data supporting the conclusions of this article will be made available by the authors, without undue reservation.

## Ethics Statement

The animal study was reviewed and approved by the Jilin University Animal Care and Use Committee.

## Author Contributions

TS, WL, DL, HM, J-YL, and JML designed the research. QM, FY, JL, QZ, DW, SX, and CC did experiments and collected data for this manuscript. J-YL, HM, QM, FY, JL, and PL analyzed the data. J-YL, KP, HM, and TS wrote the manuscript. TS, WL, DL, HM, and JML provided funding and resources to support this research. All authors contributed to the article and approved the submitted version.

## Conflict of Interest

The authors declare that the research was conducted in the absence of any commercial or financial relationships that could be construed as a potential conflict of interest.

## Footnotes

1 http://labs.gaidi.ca/mouse-brain-atlas

## Supplementary Material

Supplementary Figure 1 Unilateral saline injection at the thalamic VB complex did not modulate the distribution of cortical onset sites. **(A)** Spatial distribution of cortical burst onset sites before versus after saline injection. Each row corresponds to one animal. Saline was injected in the left thalamic VB complex. **(B)** Summary of the cortical onset laterality in the 2 animals. No significant change of onset laterality was noticed after thalamic saline injection (chi-square test, *p* = 0.455).
