# Supplementary material for: Isoflurane-Induced Burst Suppression Is a Thalamus-Modulated, Focal-Onset Rhythm With Persistent Local Asynchrony and Variable Propagation Patterns in Rats
Source: Front Syst Neurosci. 2021 Jan 12;14:599781. doi: 10.3389/fnsys.2020.599781 (PMC7835516; doi:10.3389/fnsys.2020.599781)
Supplement: Supplementary Figure 1 — Unilateral saline injection at the thalamic VB complex did not modulate the distribution of cortical onset sites. (A) Spatial distribution of cortical burst onset sites before versus after saline injection. Each row corresponds to one animal. Saline was injected in the left thalamic VB complex. (B) Summary of the cortical onset laterality in the 2 animals. No significant change of onset laterality was noticed after thalamic saline injection (chi-square test, p = 0.455). [file Table_1.DOCX]

Supplementary Material


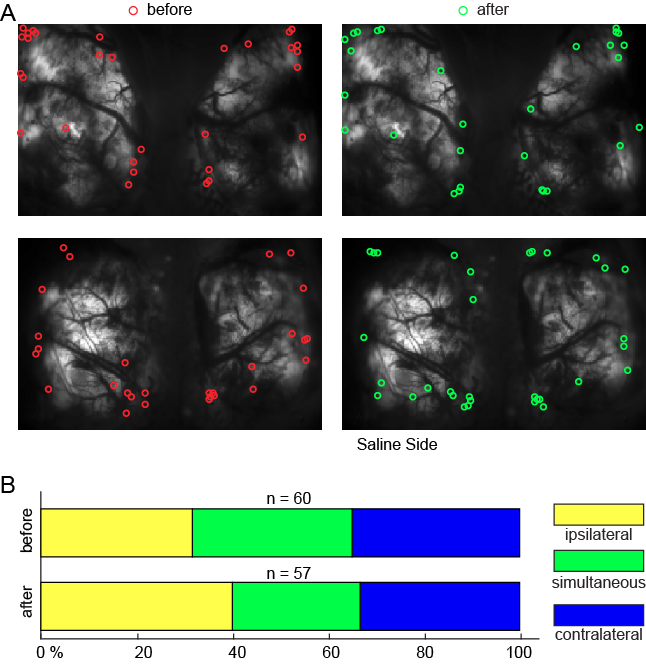


**Supplementary Figure 1. Unilateral saline injection at the thalamic VB complex did not modulate the distribution of cortical onset sites. (A)** Spatial distribution of cortical burst onset sites before versus after saline injection. Each row corresponds to one animal. Saline was injected in the left thalamic VB complex. **(B)** Summary of the cortical onset laterality in the 2 animals. No significant change of onset laterality was noticed after thalamic saline injection (chi-square test, p =0.455).
